# Prevalence Estimation of Dementia/Alzheimer’s Disease Using Health and Retirement Study Database in the United States

**DOI:** 10.14283/jpad.2024.114

**Published:** 2024-06-20

**Authors:** Amir Abbas Tahami Monfared, N. Hummel, A. Chandak, A. Khachatryan, R. Zhang, Q. Zhang

**Affiliations:** 1 Eisai, Inc., 200 Metro Blvd, Nutley, NJ 07110 USA; 2 Epidemiology, Biostatistics and Occupational Health, McGill University, Montreal, Canada; 3 Certara GmbH, Berlin, Germany; 4 Certara Inc., Radnor, USA; 5 Certara Ltd., London, UK

**Keywords:** Alzheimer’s disease, mild cognitive impairment, prevalence, severity distribution

## Abstract

**Background:**

Updated prevalence estimates along the continuum of Alzheimer’s disease (AD) can foster a more nuanced and effective approach to managing AD within the current healthcare landscape.

**Objectives:**

This study aims to estimate the prevalence and severity distribution of dementia/AD (including mild, moderate, and severe stages) and all-cause mild cognitive impairment (MCI) in the United States using data from the Health and Retirement Study (HRS).

**Design:**

Retrospective study.

**Setting:**

Data from the bi-annual HRS surveys involving in-depth interviews of a representative sample of Americans aged >50 years.

**Participants:**

Dementia/AD and all-cause MCI patients from the 4 most recent HRS surveys (2014, 2016, 2018 and 2020).

**Measurements:**

AD was identified based on diagnosis (self-report). Cognitive performance (modified Telephone Interview of Cognitive Status [TICS-m]) scores in the dementia/AD range were also captured; all-cause MCI was similarly identified using the TICS-m. Dementia/AD and MCI prevalence, as well as the distribution by dementia/AD stage (mild, moderate, or severe), were estimated. Sampling weights developed by HRS were applied to ensure the sample’s representativeness of the target population and unbiased estimates for population parameters.

**Results:**

Across the four HRS surveys, the total number of HRS respondents ranged from 15,000 to 21,000 (unweighted); 7,000 to 14,000 had TICS-m scores. The estimated prevalence of AD (all severity categories combined) in the 2014, 2016, 2018, and 2020 HRS surveys was 1.2%, 1.2%, 1.3% and 1.0%, respectively using the diagnosis-based approach; using the cognitive performance-based approach, 23–27% patients had scores in the dementia/AD ranges across the 4 surveys. The estimated prevalence of all-cause MCI was consistently 23% in each survey. In the 2020 survey, the distribution of mild, moderate, and severe disease stages was 34%, 45%, and 21%, respectively, in patients self-reporting an AD diagnosis, and 55%, 40%, and 5%, respectively in all patients meeting TICS-m threshold for dementia/AD.

**Conclusion:**

The prevalence of AD diagnosis based on self-report was approximately 1% across the 4 most recent HRS surveys and may reflect the proportion of patients who have actively sought healthcare for AD. Among HRS survey respondents with cognitive scores available, over 20% were in the dementia/AD range. The distribution of disease by stage differed for self-reported AD diagnosis vs dementia/AD based on cognitive scores. Discordance in estimates of dementia/AD and stage distributions underscores a need for better understanding of clinical practice patterns in AD diagnosis, use of clinical assessment tools, and severity classification in the United States. Accurate patient identification is needed, especially early in the AD disease continuum, to allow for timely and appropriate initiation of new anti-amyloid treatments.

## Introduction

**A**lzheimer’s disease (AD) is a progressive neurodegenerative condition estimated to affect 6.7 million Americans aged 65 or older in 2023. (1) An additional 5 to 7 million Americans aged 65 years or older may have mild cognitive impairment (MCI) due to AD (i.e., MCI that will likely progress to AD dementia) (1–4). By 2050, the numbers of people with AD are projected to almost double in the United States and Europe, and triple world-wide (5–8).

Research on the prevalence of MCI and/or of AD dementia across different severity stages is currently limited (9, 10). An early study of AD prevalence by disease severity, based on the Chicago population study, reported that in 2000, 48%, 31%, and 21% of prevalent cases were classified as mild, moderate, and severe, respectively (10). A similar distribution was reported in an analysis of the population-based Framingham Heart Study (FHS) which reported 50%, 30%, and 19% of patients with AD had mild, moderate, and severe stage disease, respectively based on pooled data in 3 time-windows (2004–2005; 2006–2007, and 2008–2009) (9). Updated prevalence estimates along the continuum of AD are needed, especially since newly available anti-amyloid treatments are indicated for initiation in early stage disease. These estimates not only serve as fundamental metrics for gauging the prevalence of AD at various disease stages, but also play a critical role in delineating the potential impact of these new therapies. A comprehensive understanding of the AD prevalence landscape can empower clinicians and other healthcare stakeholders in the AD community to make informed decisions about the implementation and implications of novel AD treatments, fostering a more nuanced and effective approach to managing AD within the healthcare landscape.

The current study was conducted to estimate the prevalence and severity distribution of all-cause MCI and mild, moderate, and severe AD in the United States using data from the Health and Retirement Study (HRS).

## Methods

### Data source

This retrospective analysis utilized data from HRS (11, 12), a longitudinal panel study that was initiated in 1992 by the University of Michigan. HRS uses in-depth interviews to survey a representative sample of over 20,000 Americans aged greater than 50 years bi-annually, with a new cohort of individuals aged 51–56 added every 6 years (13). The current investigation analyzed the 4 most recent HRS surveys (2014, 2016, 2018 and 2020).

### Study sample

Patients with AD were identified using a diagnosis-based approach. A cognitive performance-based approach was used to capture additional patients who may have had scores in the dementia/AD range. The diagnosis-based approach identified respondents as having AD if they answered “yes” to the initial survey question, “Has a doctor ever told you that have Alzheimer’s Disease?” or “yes” to the repeat survey question, “Since we last talked to you, has a doctor told you that you have Alzheimer’s Disease?” The cognitive performance-based approach identified respondents if they were considered as having dementia/AD or all-cause MCI based on their cognitive performance (test score), at or before the considered time points. Since amyloid status was unknown, cases identified using cognitive scores alone are referred to as “dementia/AD” rather than “AD” specifically. Cognitive performance was assessed by the modified Telephone Interview of Cognitive Status (TICS-m), which is part of the bi-annual interviews in the HRS. Since TICS-m does not have established cut-offs for categorizing HRS respondents into MCI or mild, moderate, and severe dementia/AD, a crosswalk to the mini-mental state examination (MMSE) (14) method was applied as described previously (15). Based on this method, an adjusted TICS-m score of 20–22 corresponds to MCI, while education-adjusted TICS-m scores of 17–19, 10–16 and 0–9 correspond to mild, moderate, and severe dementia/AD, respectively.

Each survey collected self-reported AD diagnosis data from the prior 2 years (i.e., 2014 included 2013 and 2014 data), whereas cognitive test score data for dementia/AD were only collected cross-sectionally in the survey years (i.e., 2014, 2016, 2018, and 2020). Patients with all-cause MCI were identified based on their cognitive performance only.

### Prevalence estimation

The prevalence of AD was estimated using a diagnosis-based approach by dividing the number of patients with a self-reported diagnosis of AD by all survey respondents. In addition, to estimate the proportion of patients with cognitive scores in the MCI or dementia/AD ranges, the number of patients with TICS-m corresponding to MCI or dementia/AD was divided by the number of survey respondents for whom TICS-m scores were available.

The distribution by dementia/AD stage (mild, moderate, or severe) among patients identified through each of the 2 approaches was assessed by survey year.

### Statistical analysis

The respondents’ baseline characteristics were summarized using descriptive statistics, using number of observations (n) and percentage (%) for categorical variables and mean and standard deviation (SD) for continuous variables.

If subitems of the TICS-m were missing, a multivariate, regression-based imputation, using a combination of relevant demographic, health, and economic variables as well as cognitive variables from prior and current surveys, was applied; this method has been described previously and shows that 4.3%, 4.8%, 3.2%, and 3.9% of all HRS respondents had a least one imputed cognitive score in the 2014, 2016, 2018, and 2020 surveys, respectively (16). HRS sampling weights were applied to all variables; development of those sampling weights is described elsewhere (17). In brief, HRS developed sampling weights for survey respondents which are used to account for differential probabilities of selection, non-response, and institutionalization status. The weights, determined through a combination of post-stratification, ranking ratio estimation, and calibration methods, are designed to ensure the sample’s representativeness of the target population, facilitating the production of unbiased estimates for population parameters.

## Results

### Study Population: Weighted Demographics

Across the four HRS surveys, the total number of HRS respondents ranged from approximately 15,000 to 21,000 (unweighted); the number of respondents with available TICS-m scores ranged from approximately 7000 to 14,000 (unweighted). Weighted patient characteristics in each of the HRS surveys are presented in Table 1. Using the diagnosis-based approach, approximately

**Table 1:** Patient Characteristics in Each of 4 Bi-Annual HRS Surveys: Weighted Demographics

**Total HRS respondents**		**2014**	**2016**	**2018**	**2020**
		**N=18,747**	**N=20,912**	**N=17,146**	**N=15,723**
*(1) Identified with diagnosis-based approach (self-report)*					
		*n=337*	*n=311*	*n=251*	*n=215*
AD	Age, mean (SD)	81.3 (9.3)	80.7 (10.9)	80.0 (10.4)	79.1 (10.0)
	Female, %	63%	63%	61%	61%
	Completed college, %	21%	20%	28%	23%
Total HRS respondents with TICS-m score		N=9692	N=13,700	N=7817	N=7361
*(2) identified with cognitive performance-based approach (cognitive scores)*					
		*n=2974*	*n=3730*	*n=2272*	*n=2038*
Dementia/AD	Age, mean (SD)	78.0 (8.5)	73.2 (12.4)	77.8 (8.5)	77.8 (8.5)
	Female, %	55%	53%	54%	55%
	Completed college, %	24%	27%	26%	27%
		*n=2329*	*n=3268*	*n=1870*	*n=1672*
All-cause MCI	Age, mean (SD)	74.5 (7.6)	68.4 (11.4)	74.7 (7.7)	74.8 (7.4)
	Female, %	53%	49%	51%	53%
	Completed college, %	30%	35%	34%	38%

AD, Alzheimer’s disease; MCI, mild cognitive impairment; SD, standard deviation. Unweighted N/n values are shown.

### Prevalence of Dementia/AD and All-cause MCI: Weighted Estimates

The overall prevalence of AD (all severity categories combined), using the diagnosis-based approach, was estimated at 1.2%, 1.2%, 1.3% and 1.0% in 2014, 2016, 2018 and 2020 HRS surveys, respectively (Table 2). Based on cognitive performance, the proportions of patients with scores meeting the threshold for dementia/AD were 27%, 22%, 24% and 23% in 2014, 2016, 2018 and 2020 HRS surveys, respectively, while the proportion with scores categorized as all-cause MCI was consistently estimated to be 23% in each of the HRS surveys.

**Table 2:** Prevalence of Dementia/AD and All-Cause MCI in Each Bi-annual HRS Survey: Weighted Estimates

**(1) Identified with diagnosis-based approach (self-report)**				
	**2014**	**2016**	**2018**	**2020**
AD prevalence	1.2%	1.2%	1.3%	1.0%
**(2) Identified with cognitive performance-based approach (cognitive scores)**				
	**2014**	**2016**	**2018**	**2020**
Dementia/AD prevalence	27%	22%	24%	23%
All-cause MCI prevalence	23%	23%	23%	23%

AD, Alzheimer’s disease; MCI, mild cognitive impairment; Each respondent is only represented once per survey.

### Distribution of Disease Severity by Stage Among Patients with AD or Dementia/AD

**Figure 1:**
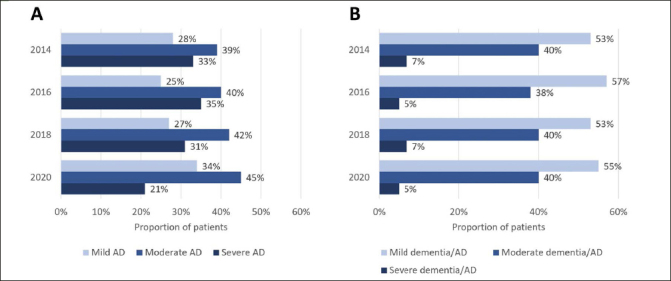
Distribution of disease severity by stage in each HRS survey using (A) diagnosis-based identification (self-reported AD)^a^; (B) cognitive performance-based identification (cognitive scores corresponding to dementia/AD)^b^ AD, Alzheimer’s disease; a. Among HRS patients who self-reported AD, TICS-m scores were available for 92/337 (27%), 89/311 (29%), 80/251 (32%), and 68/215 (32%) in the 2014, 2016, 2018, and 2020 surveys, respectively; b. Among all HRS patients, TICS-m scores were available for 9692/18,747 (52%), 13,700/20,912 (66%), 7817/17,146 (46%), and 7361/15,723 (47%) in the 2014, 2016, 2018, and 2020 surveys, respectively.

Among HRS respondents with diagnosis-based AD who had TICS-m scores available for stage categorization, the proportion with mild AD ranged from 25% in the 2016 survey to 34% in the 2020 survey (Figure 1A). The greatest proportion of respondents had moderate AD, ranging from 39% in the 2014 survey to 45% in the 2020 survey. The proportion of respondents with severe AD ranged from 21% in the 2020 survey to 35% in the 2016 survey.

Among all patients who met the cognitive score threshold for dementia/AD, over half were categorized as mild, ranging from 53% in the 2014 survey to 57% in the 2016 survey (Figure 1B). The proportion of patients with cognitive scores indicative of moderate dementia/ AD ranged between 38% in the 2016 survey and 40% in the other HRS surveys, while the proportion of patients classified as severe dementia/AD was consistently ≤7% in each of the HRS surveys that were analyzed.

## Discussion

The current study analyzed the 4 most recent bi-annual HRS surveys (2014, 2016, 2018, 2020) to provide prevalence estimates of dementia/AD and severity distribution in the United States. A diagnosis-based (self-reported) approach was used for identifying AD in the HRS population. Additional cases of dementia/AD were captured using a cognitive performance-based approach (using TICS-m scores). The proportion of patients with cognitive test scores corresponding to dementia/AD (mild, moderate, and severe stages combined) were consistently about 20 times higher than estimates of diagnosed AD in each of the HRS surveys (e.g., 23% vs 1% in the 2020 survey). Cognitive scores were not specific for AD (identified AD as well as other dementias); however, inclusion of all-cause dementia alone would not account for the markedly higher estimates relative to diagnosis-based estimates. We speculate that patients with self-reported AD likely reflect the volume of patients who have sought healthcare for AD; whereas cognitive testing may reveal additional patients who are living with dementia/AD but have not yet been identified as such within the healthcare system.

Using cognitive performance, 23% of patients had cognitive scores corresponding to all-cause MCI in each of 4 HRS surveys. This is comparable to the 22% prevalence of all-cause MCI reported previously using the Harmonized Cognitive Assessment Protocol (HCAP), a cross-sectional nationally representative subsample of HRS in 2016 (2).

The distribution of mild, moderate, or severe AD severity stage differed between patients self-reporting AD and those with cognitive scores corresponding to dementia/AD in each of the surveys. There were markedly lower proportions of mild and higher proportions of severe stage disease among those with self-reported AD vs those meeting the TICS-m threshold for dementia/AD. For example, in the 2020 survey, the distribution of mild, moderate, and severe stages was 34%, 45%, and 21%, respectively among patients reporting an AD diagnosis and 55%, 40%, and 5%, respectively among patients with TICS-m scores meeting the threshold for dementia/AD. Our cognitive performance-based finding that approximately half of dementia/AD cases in HRS were in the mild stage is consistent with prior investigations of AD severity stage distribution in the United States from HCAP and FHS (9, 10). In contrast, our findings regarding the distribution of other moderate and severe stages differ from the distributions reported in these earlier investigations: the proportion of patients with moderate stage disease was 38-45% in our investigation (across the 4 HRS surveys for both diagnosed and cognitive-score based cases) vs approximately 30% in the HCAP- and FHS-based investigations; the proportion with severe disease in our analysis was comparable or higher (21–35%) among those with an AD diagnosis, but notably lower (5–7%) among those with dementia/AD based on cognitive scores, than in prior investigations (approximately 20%) (9, 10). We can only speculate that not all the cognitive assessment instruments are equally sensitive across the spectrum of disease severity. There is a possibility that TICS-m may be less sensitive for assessing patients with severe AD. Further, it is possible that people with severe dementia may be less likely to complete self-report, suggesting that the percentage of severe AD among self-reported patients may not be reflective of a truly severe stage of disease. Other factors that may contribute to disparate severity distributions may include differences in study designs, population characteristics, and diagnostic criteria. For instance, the HRS is a nationally representative study of adults aged 50 and older (11, 13), while the FHS is a community-based study of adults (original cohort aged 30–62 years) from a single town in Massachusetts (9, 18). Moreover, the diagnostic criteria used to define AD have evolved over time (19, 20), which could also contribute to differences in prevalence estimates across studies, particularly by disease severity. Also of interest, although not specific to AD, the Dementia United Kingdom 2014 Expert Consensus reported the severity stage distribution among patients with late-onset dementia to be approximately 55%, 32%, and 13% for mild, moderate, and severe disease, respectively (21).

Of note, a systemic review of studies estimating cognitive impairment in adults aged 50 years or older found that, among studies that used cognitive tests (e.g., MMSE), the prevalence of cognitive impairment ranged from 5.1% to 41%. In our analysis, TICS-m identified some cognitive impairment in 45–50% of patients (combining MCI and dementia) across the 4 surveys; these higher rates in our analysis could, in part, be due to the HRS including some patients in nursing home settings, whereas the studies analyzed in the systematic review were limited to the community setting (22).

### Limitations

Self-report of AD in the HRS may reflect the patients who have sought care for AD in the healthcare system, but likely underestimates the total number of patients living with AD, particularly in early stages of disease. Stigma associated with AD or lack of knowledge about AD, which are known barriers to diagnosis of AD (23), as well as healthcare access-related barriers could all contribute to lower rates of AD by self-report. Notably, even when patients have adequate access to care, cognitive assessments may not be done as part of their visits. In addition, even some clinicians may be hesitant to assign an AD diagnosis due to concerns about stigmatization (24). Discordance between clinician judgement-based vs cognitive score-based assessment of dementia/AD has been documented previously, leading the investigators to speculate that a possible reason for this discordance is that clinicians consider psychiatric, behavioral, and functional factors in addition to cognitive factors in their patient assessments (25). Additionally, data collection using telephone surveys could be impacted by factors such as participant capability or hearing issues. Another potential limitation is that, across the 4 surveys, many HRS respondents had missing TICS-m scores (34–54% of all respondents; 68–73% of respondents self-reporting AD); among patients already aware of their AD diagnosis, there may be a lower interest in engaging with the TICS-m, or a reduced capability to answer TICS-m questions. Use of imputed cognition values for missing responses may have influenced study findings; however, this imputation method was applied to be consistent with prior HRS studies, and to reduce potential bias associated with excluding a large portion of cognitively impaired participants as they had a greater proportion of missing data. Finally, categorizing patients as MCI and/or dementia/AD using TICS-m relied on test scores that were benchmarked using the MMSE; however, this approach has not been validated. Without information regarding amyloid status, use of cognitive tests cannot determine whether dementia is due to AD specifically; similarly test scores alone can classify patients with MCI, but cannot discern MCI due to AD from all-cause MCI. Of note, positive amyloid positron emission tomography results have been reported in 55% of MCI cases and 70% of dementia cases (4).

The study population, derived from the HRS study, is both large and nationally representative. However, the prevalence estimates, which rely on self-reporting of AD, predominantly reflect individuals actively seeking healthcare. Nevertheless, self-reported data offer a unique perspective on disease prevalence, albeit differing from estimates based on comprehensive case ascertainment methods. Still, such data remain valuable in illustrating current healthcare demand, bearing considerable implications for health policy.

## Conclusion

The prevalence of AD diagnosis based on self-report was approximately 1% across the 4 most recent HRS surveys, and may reflect the proportion of patients who have actively sought healthcare for AD. Among HRS survey respondents with cognitive scores available, over 20% had scores in the dementia/AD range. The distribution of disease by stage differed when considering patients with a self-reported AD diagnosis vs those meeting the threshold for dementia/AD based on cognitive performance: 34% vs 55% mild, 45% vs 40% moderate, and 21% vs 5% severe in the most recent (2020) survey. The discordance in these estimates and stage distributions underscores a need for better understanding of clinical practice patterns in AD diagnosis, use of clinical assessment tools, and severity classification in the United States. Accurate patient identification is needed, especially early in the AD disease continuum, to allow for timely and appropriate initiation of new anti-amyloid treatments.

## Notes

*Ethical standards:* The HRS has been approved by the Institutional Review Board at the University of Michigan. The HRS obtains informed verbal consent from voluntary participants and follows strict procedures to protect study participants from disclosure (including maintaining a Federal Certificate of Confidentiality). The public data, made available under restricted data use agreements to researchers and used in this study, is de-identified. The authors who worked on the data analysis agreed and adhered to the conditions of the data use agreement set forth by HRS.
